# Image thresholding segmentation based on weighted Parzen-window and linear programming techniques

**DOI:** 10.1038/s41598-022-17818-4

**Published:** 2022-08-10

**Authors:** Fusong Xiong, Zhiqiang Zhang, Yun Ling, Jian Zhang

**Affiliations:** 1 Soochow College, Soochow University, Suzhou, 215006 Jiangsu China; 2 Collaborative Innovation Center of Novel Software Technology and Industrialization, Nanjing University, Nanjing, 210008 Jiangsu China; 3 Wenzheng College, Soochow University, Suzhou, 215104 Jiangsu China

**Keywords:** Computer science, Software

## Abstract

Image segmentation by thresholding is an important and fundamental task in image processing and computer vision. In this paper, a new bi-level thresholding approach based on weighted Parzen-window and linear programming techniques is proposed to use in image thresholding segmentation. First, by proposing a weighted Parzen-window to describe the gray level distribution status, we obtain the boundaries for the foreground and background of the image. Then the image thresholding problem can be transformed into the problem of solving a linear programming problem for computing the coefficient values of weighted Parzen-window. The results of testing on synthetic, NDT and a set of benchmark images indicate that the proposed method can achieve a higher segmentation accuracy and robustness in comparison to some classical thresholding methods, such as inter class variance method (OTSU), Kapur’s entropy-based method (KSW), and some state-of-art methods that consider spatial information, such as CHPSO, GLLV histogram method and GABOR histogram method.

## Introduction

Image segmentation technology has been widely applied in industry, agriculture, military fields, etc. Among all the image segmentation methods, thresholding is one of the most widely used, because it is simple and ease to implement. Image thresholding methods are divided into two categories, one is bi-level thresholding methods and the other is multi-level methods. The bi-level methods that involve the fundamental assumption that the foregrounds and backgrounds of the image have different gray level distributions, segment the image to foregrounds and backgrounds. The multi-level methods can be generalized by the bi-level methods that segment the image to multiple non-overlapping regions^1^. So far, many successful thresholding methods have been developed and applied in many fields, such as infrared nondestructive testing, magnetic resonance imaging, etc.^2^. In this paper, we focus on bi-level thresholding methods.

In the process of bi-level thresholding, it is assumed that there exists an optimal threshold value separating the gray levels. The segmentation task can be implemented by classifying pixels whose gray level is less than the threshold as backgrounds and pixels whose gray level is greater than the threshold as foregrounds, or vice versa. For decades, some classical bi-level thresholding algorithms have been proposed, such as the inter-class variance method (OTSU)^3^, minimum error bi-level thresholding method (MET)^4^, the entropic bi-level thresholding method based on one-dimensional histogram (1D KSW)^5^, Renyi’s entropic bi-level thresholding method^6^ and Tsallis’s entropic bi-level thresholding method^7^ etc. In addition, these classical methods have been modified or combined with other techniques to develop numerous successful bi-level or multi-level thresholding methods. As typical examples, relative entropy theory and 3D histogram were combined with MET for an optimal threshold discriminant^8^. The ant colony optimization approach was combined with the inter-class variance method for fast find out multiple thresholds of the images^9^. The hybrid whale optimization approach was combined with the 1D KSW method for multi-level thresholding segmentation^10,11^. The meta-heuristics approach was combined with Renyi’s entropy-based method for multi-level thresholding segmentation^12^. The particle swarm optimization approach was combined with the Tsallis entropy-based method for multi-level thresholding segmentation^13^. The convergence heterogeneous particle swarm optimization algorithm, was utilized to find the optimal bi-level and multi-level thresholds^14^. The coyote optimization algorithm, which takes Ostu and fuzzy entropy as objective functions, was used to multi-level thresholds selection^15^. Although many thresholding methods have been developed, the entropy-based methods remain the most popular. Many extensions of the entropy-based method, which are based on 1D histogram, have been proposed in recent years. For examples, Cheng proposed a new bi-level thresholding method by implementing fuzzy segmentation based on two-dimensional (2D) histograms^16^. Xiao proposed two new entropic bi-level thresholding methods. The first method employs gray level spatial correlation (GLSC) histogram^17^. In contrast to the 2D histogram, the GLSC histogram is obtained using the gray level of the pixels and their neighbors with similar gray level. The second method employs gray level and gradient magnitude (GLGM) histogram^18^. The GLGM histogram clearly captures the occurrence probability and spatial distribution features of gray level at the same time, and considers spatial information. Utilizing the orientation histogram of a gradient image to calculate the local edge property, a new bi-level thresholding method employing 2D-D histogram was proposed by Yimit^19^. A new thresholding method based on a GLLV histogram was proposed by Zheng^20^ using the gray level information of pixels and its local variance in a neighborhood. A new thresholding method based on a GABOR histogram was proposed by Yi^21^. Recently, Xiong et al. proposed a new image thresholding method combining Kapur’s entropy with Parzen-window estimation^22^.

In general, the improved 2D histogram methods outperform 1D histogram methods. However, the 2D entropic thresholding methods still have some limitations, such as, not a generic method for image thresholding, and lack of robustness or stability etc.

**Figure 1:**
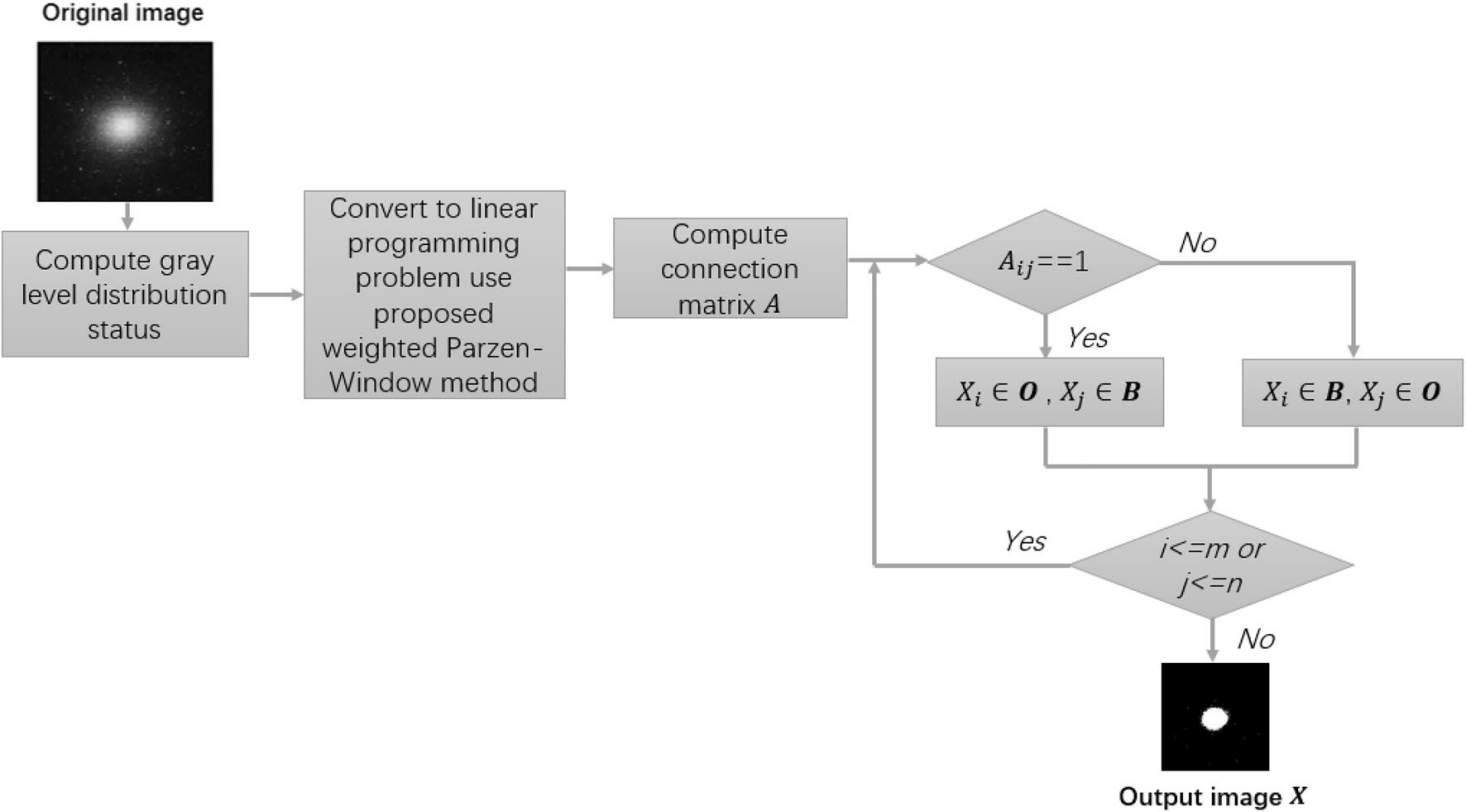
Process of the proposed thresholding method.

In this paper, we try to propose a new bi-level thresholding method, which is based on the boundaries for the foreground and background by using a weighted Parzen-window to describe the gray level distribution status rather than gray level probability density distributions (1D or 2D histogram) for the foreground and background in an image. Subsequently, image thresholding was successfully transformed into a linear programming problem. We used the simplex method to solve the linear programming problem. In the experimental section, the proposed method is compared with the classic and state-of-art methods to demonstrate its accuracy and robustness. The novel contribution of this study is the construction of a new data distribution description method based on the weighted Parzen-window technique, which can be regarded as a linear programming problem. This process is illustrated in Fig. 1.

The rest parts of this paper is organized as follows. In section "The proposed method", we briefly introduce the Parzen-window technology, and provide a new bi-level thresholding method based on the weighted Parzen-window and linear programming. In section "Experimental results", the results of the experiments and a discussion are presented. Finally, section "Conclusions" gives the conclusion.

## The proposed method

### Parzen-window technique and its use in image estimation

For a gray image $F=\left\{f\left(x,y\right)|x\in \left\{\mathrm{1,2},3,\dots ,m\right\}, y\in \left\{\mathrm{1,2},3,\dots ,n\right\}\right\}$ of size $m\times n$ with $L$ gray levels, the gray level set $G=\left\{\mathrm{0,1},2,\dots ,L-1\right\}$*.* $f\left(x,y\right)\in G$ is the gray value of the pixel located at location $\left(\mathrm{x},\mathrm{y}\right)$. Let ${\omega }_{l}=\left\{\left(x,y\right) |f\left(x,y\right)=l, x\in \left\{\mathrm{1,2},3,\dots ,m\right\},y\in \left\{\mathrm{1,2},3,\dots ,n\right\}, l\in G\right\}$,${C}_{l} \left(l\in G\right)$ represents the number of pixels in ${\omega }_{l}$ , then $\omega =\left\{ {\omega }_{l}, l\in G\right\}$ and $N=\sum_{l=0}^{L-1}{C}_{l}$ . Obviously, ${\omega }_{l}$ is defined in 2D space. Suppose that $t$ is the threshold value, the result of bi-level thresholding by $\mathrm{t}$ is a binary function ${f}_{t}\left(x,y\right)$:

1 $${f}_{t}\left(x,y\right)=\left\{\begin{array}{*{20}l}0 & \quad if \,f\left(x,y\right)\le t\\ 1 & \quad if \,f\left(x,y\right)>t\end{array}\right.$$

The result of thresholding is a clustering problem that separates all pixels into two classes $O$ and $B$. Where $O$ represents foregrounds and $B$ represents backgrounds, or vice versa.

Traditional thresholding methods are first used to compute the probability of each gray level distribution. Then, the optimal threshold value was computed by optimizing an appropriate objective function, which was designed using the gray level distribution or other properties.

As we known well, the Parzen-window estimation is an effective non-parametric estimation with solid theoretical foundation, which can better describe the distributions of data^23–25^. The basic idea is to estimate the $pdf$ using the mean value of the densities of each point within a certain range. If we want to estimate the $pdf$ at point $X$, we can place a window of size $h$ at $X$ and see how many observations of ${X}_{i}$ fall into this window. The value of $pdf$ is the average of the observations falling into this window. The Parzen-window estimate ${P}_{n}\left(X\right)$ can be expressed as:

2 $${P}_{n}\left(X\right)=\frac{1}{n}\sum_{i=1}^{n}\frac{1}{{V}_{n}}K\left(X-{X}_{i}\right)$$

where ${V}_{n}$ is the volume of the d-D hypercube with edge length ${h}_{n}$, ${V}_{n}={h}_{n}^{d}$, ${h}_{n}=\frac{c}{\sqrt{n}}$ is called window width. $\mathrm{c}$ is a constant parameter that always takes the value of 1. $K\left(\cdot \right)$ is the d-D kernel function (window function), and:

3 $$\int K\left(X\right)dX=1$$

The most commonly used kernel function is the Gaussian kernel function (normal distribution), defined as:

4 $$K\left(X-{X}_{i}\right)=\frac{1}{{\left(\sqrt{2\pi }\right)}^{d}}exp\left(-\frac{{\left(X-{X}_{i}\right)}^{2}}{2{{h}_{n}}^{2}}\right)$$

Following the Parzen-window estimation, for the 2-D image $F$, the sample $\left(x,y\right)$ in the two-dimensional point space ${\omega }_{l}$, its $pdf$ $p\left(x,{y,\omega }_{l}\right)$ can be estimated by Eqs. (5) and (6).

5 $$p\left(x,y|{\omega }_{l}\right)=\frac{1}{{C}_{l}}\sum_{j=1}^{{C}_{l}}\frac{1}{{V}_{{C}_{l}}}\varphi \left(x,y;{x}_{j,}{y}_{j}\right)$$

6 $$p\left(x,{y,\omega }_{l}\right)=p\left({\omega }_{l}\right)p\left(x,y|{\omega }_{l}\right)$$

where ${C}_{l}$ is the number of pixels in ${\omega }_{l}$, $p\left({\omega }_{l}\right)$ can be approximated by a histogram, given by:

7 $$p\left({\omega }_{l}\right)=\frac{{C}_{l}}{N}, l\in \left\{\mathrm{0,1},2,\dots ,L-1\right\}$$

where $N=\sum_{l=0}^{L-1}{C}_{l}$. Then, the $p\left(x,y,{\omega }_{l}\right)$ is obtained by:

8 $$\begin{aligned}p\left(x,y,{\omega }_{l}\right)&=p\left({\omega }_{l}\right)p\left(x,y|{\omega }_{l}\right)=\frac{{C}_{l}}{N}\cdot \frac{1}{{C}_{l}}\sum_{j=1}^{{C}_{l}}\frac{1}{{V}_{{C}_{l}}}\varphi \left(x,y;{x}_{j},{y}_{j}\right)\\ &=\frac{1}{N}\sum_{j=1}^{{C}_{l}}\frac{1}{{V}_{{C}_{l}}}\varphi \left(x,y;{x}_{j},{y}_{j}\right)\end{aligned}$$

where $\left({x}_{j},{y}_{j}\right)$ denotes the coordinates of $jth$ sample (pixel) in ${\omega }_{l}$, ${V}_{{C}_{l}}$ represents the volume of the cube whose edge length is ${\sigma }_{l}$, ${\sigma }_{l}$ is also called the window width, that is, for a two-dimensional image, ${V}_{{C}_{l}}={{\sigma }_{l}}^{2}$. $\varphi \left(\cdot \right)$ is a window function (also called kernel function). We chose Gaussian kernel function, which is taken as:

9 $$\varphi \left(x,y;{x}_{j},{y}_{j}\right)=\frac{1}{{\left(\sqrt{2\pi }\right)}^{2}}exp\left(-\frac{{\left(x-{x}_{j}\right)}^{2}+{\left(y-{y}_{j}\right)}^{2}}{2{{\sigma }_{l}}^{2}}\right)$$

Thus, $p\left(x,y\right)$ can be estimated using:

10 $$\begin{aligned}p\left(x,y\right)&=\sum_{l=0}^{L-1}p\left(x,{y,\omega }_{l}\right)=\sum_{l=0}^{L-1}p\left({\omega }_{l}\right)p\left(x,y|{\omega }_{l}\right)\\ &=\sum_{l=0}^{L-1}\frac{1}{N}\sum_{j=1}^{{C}_{l}}\frac{1}{{V}_{{C}_{l}}}\varphi \left(x,y;{x}_{j},{y}_{j}\right)\\ &=\frac{1}{2\pi N}\sum_{l=0}^{L-1}\sum_{j=1}^{{C}_{l}}\frac{1}{{{\sigma }_{l}}^{2}}exp\left(-\frac{{\left(x-{x}_{j}\right)}^{2}+{\left(y-{y}_{j}\right)}^{2}}{2{{\sigma }_{l}}^{2}}\right)\end{aligned}$$

However, probability density estimation itself is an ill-posed problem. Moreover, the estimation of the probability density function involves a large amount of calculation, and it is easily affected by noise and the number of samples. To avoid these negative effects, we give up the estimation of probability density. We attempt to obtain the boundaries for the foregrounds and backgrounds in an image by using a weighted Parzen-window, to obtain a good description of the gray level distribution status, the thresholding problem can be converted to the problem of solving a linear programming problem for determining the coefficient values of the weighted Parzen-window.

### Weighted Parzen-window combines linear programming for image thresholding

Here, we propose the weighted Parzen-window method, which is an improvement of the Parzen-window method. By combining the proposed weighted Parzen-window method and a linear programming technique, we provide a new image thresholding method.

As is well know, thresholding segmentation assumes that the pixels are divided into two classes ${\varvec{O}}$ and ${\varvec{B}}$. If we can choose an suitable $\rho$ to divide $\left\{{\omega }_{l}, l\in G, G=\left\{\mathrm{1,2},\dots ,.L-1\right\}\right\}$ into two classes, such as $\left\{{\omega }_{O}, O\in G\right\}$, $\left\{{\omega }_{B},B\in G\right\}$, ${\omega }_{O}\bigcap {\omega }_{B}=\phi , {\omega }_{O}\bigcup {\omega }_{B}=G$, and satisfying:

11 $${\varvec{O}}{:}\,\boldsymbol{ }\boldsymbol{ }\boldsymbol{ }\boldsymbol{ }\boldsymbol{ }\boldsymbol{ }p\left(x,{y|\omega }_{i}\right)\ge \rho , \forall i\in {\omega }_{O}$$

12 $${\varvec{B}}{:}\,\boldsymbol{ }\boldsymbol{ }\boldsymbol{ }\boldsymbol{ }\boldsymbol{ }\boldsymbol{ }p\left(x,{y|\omega }_{j}\right)<\rho , \forall j\in {\omega }_{B}$$

Then, Eq. (13) can be seen as the boundary of foregrounds and backgrounds:

13 $$\widehat{p}\left(x,y\right)=\rho$$

According to the boundary, it is easy to divide the gray level into two classes. However, this approach is not always effective, because the Parzen-window technique does not provide a method for choosing an appropriate $\uprho$. Thus, the Parzen-window technique must be modified so that it can better describe the boundary of the data distribution and obtain the appropriate $\uprho$. We now provide a solution strategy.

Suppose that a d-D pattern space with $N$ samples is as follows:

14 $$X=\left\{{{X}_{1},{X}_{2},\dots ,X}_{N}\right\}, \forall {X}_{i}\in {R}^{d}, i\in I$$

where $I$ denotes the coordinate set. Now, let’s consider the following linear programming (LP).

$$\mathrm{max}\rho$$

15 $$s.t.\left\{\begin{array}{l}\sum_{i=1}^{N}{a}_{i}=1, \\ \sum_{i=1}^{N}{a}_{i}\varphi \left({X}_{k},{X}_{i}\right)\ge \rho , \forall k\in I \\ {a}_{i}\ge 0, \forall i\in I\end{array}\right.$$

where $\varphi \left(\cdot \right)$ denotes the kernel function. Because the kernel function $\varphi \left(\cdot \right)$ and coefficient ${a}_{i}$ are non-negative, the implicit constraint is $\rho \ge 0$. The solution of the above LP and the kernel function together constitute a new description of the data distribution:

16 $$\widehat{p}\left(X\right)=\sum_{i\in {I}^{^{\prime}}}{a}_{i}\varphi \left(X,{X}_{i}\right)$$

where ${I}^{^{\prime}}=\left\{i|i\in I and {a}_{i}>0\right\}$. The solution of Eq. (15) is guaranteed by the following theorem.

#### Theorem 1

The solution of Eq. (15) is absolute existence.

#### Proof

According to the constraints in Eq. (15), we have:

17 $${0\le a}_{i}\le 1, \forall i\in I\quad \mathrm{ and }\quad 0\le\uprho \le \frac{1}{{\left(\sqrt{2\pi }\sigma \right)}^{d}}$$

It is easy to know that Eq. (15) is a feasible solution.

18 $${a}_{i}=\left\{\begin{array}{*{20}l}1, &\quad i=1 \\ 0,&\quad otherwise\end{array}\right., \quad \rho =\underset{i\in I}{\mathrm{min}}\varphi \left(X,{X}_{i}\right)$$

Thus, Eq. (15) exist in the solution domain. It can be concluded from the optimization theory that the solution of Eq. (15) is an absolute existence. Proof end.

If $\varphi \left({X}_{j},{X}_{i}\right)$ is regarded as a measure of the similarity between samples $j$ and $i$, Eq. (15) provides a strategy for selecting $\uprho$. The constraints of Eq. (15) make the inter-class similarity as large as possible. Therefore, the boundaries of the data distribution can be better delineated. Simultaneously, $\widehat{p}\left(X\right)$ is not the probability density estimation, instead, focus on describing the boundaries of data distribution, and:

19 $$\sum_{i=1}^{N}\widehat{p}\left({X}_{i}\right)=1$$

The simplex method^26^ is the most commonly used method to solve the LP problem, thus, we chose it for this study.

A gray image is regarded as a two-dimensional sample space. This can be easily mapped to linear programming. For example, a 2-dimensional space $X$ is replaced by $\left\{f\left(x,y\right)|x\in \left\{\mathrm{1,2},3,\dots ,m\right\},y\in \left\{\mathrm{1,2},3,\dots ,n\right\}\right\}$ , the index coordinate $I$ is replaced by the pixel coordinate set $\omega$, kernel function $\varphi \left(\cdot \right)$ is the same as in Eq. (9). Thus, we can classify all gray levels into two classes using the proposed weighted Parzen-window and linear programming based image thresholding (WPWLPT) method.
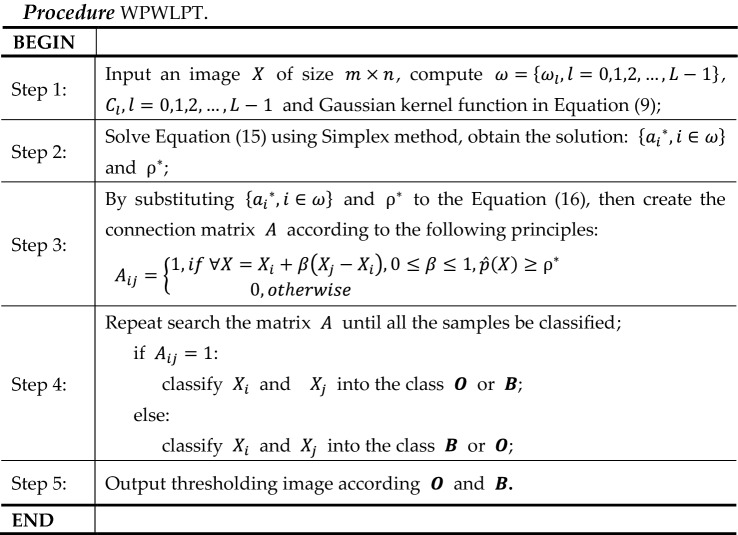


## Experimental results

In this section, we present the experimental results, obtained by using some classic methods (such as OTSU^3^ and KSW^5^), some state-of-art methods (such as CHPSO^14^, GLLV^19^ and GABOR^20^, and all the parameters being set to the default values during the experiments) and our proposed method (we call it as WPWLPT from now). Li et al.^14^ proposed the CHPSO method, which can be used for both bi-level and multi-level thresholding (they provide equations for both the bi-level and multi-level cases). It uses OTSU and KAPUR as objective functions, which we denote CHPSO_otsu and CHPSO_ksw, respectively.

In order to assess the effectiveness of the proposed method, we qualitatively and quantitatively assessed on lots of images. For brevity, we only reported 22 representative thresholding results, which included two synthetic, eight nondestructive testing (NDT) and a set of benchmark images. These images had different sizes and histogram types. We designed two synthetic images. The NDT images were obtained from^2^. The benchmark images belong to the Image Processing Standard Database (http://www.imageprocessingplace.com/root_files_V3/image_databases.htm) and the USC-SIPI Image Database (http://sipi.usc.edu/database/), which are well-known and widely used in the image thresholding literatures.

Currently, there are several measurements^2,21,27–29,32^ to quantitatively evaluate the quality of the image thresholding method. We used the misclassification error ($ME$)^2^, region nonuniformity ($NU$)^2^, feature similarity ($FSIM$)^29^ and mean intersection over union ($mIoU$)^32^ to quantitatively assess the different thresholding methods.

$ME$ measurement reflects the incorrect classification of foregrounds pixels to the backgrounds or vice versa^2^. For the bi-level image thresholding problem, $ME$ can be taken as:

20 $$ME=1-\frac{\left|{B}_{o}\cap {B}_{T}\right|+\left|{F}_{o}\cap {F}_{T}\right|}{\left|{B}_{o}\right|+\left|{F}_{o}\right|}$$

where ${B}_{\mathrm{o}}$ and ${F}_{\mathrm{o}}$ denote the backgrounds and foregrounds of the optimal thresholded image, ${B}_{T}$ and ${F}_{T}$ denote the backgrounds and foregrounds region pixels of the original image, and $\left|*\right|$ denotes the cardinality of the set $*$. Obviously, $ME$ equals to 1 for the worst case and 0 for the best case. $ME$ is the easiest and most effective method for the discrepancy measure^30^.

$NU$ measures the intrinsic quality of the segmented regions, is defined as:

21 $$NU=\frac{\left|{F}_{T}\right|}{\left|{B}_{T}+{F}_{T}\right|}\frac{{{\sigma }_{f}}^{2}}{{\sigma }^{2}}$$

where ${\sigma }^{2}$ denotes the variance of the image, and ${{\sigma }_{f}}^{2}$ denotes the variance of the foregrounds. ${B}_{T}$ and ${F}_{T}$ denote the backgrounds and foregrounds region pixels of the original image. Obviously, $NU$ closes to 0 for a well-segmented image and equals to 1 for the worst-segmented image.

$FSIM$ calculates the similarity of two images, is defined as:

22 $$FSIM=\frac{{\sum }_{X\in\Omega }{S}_{L}\left(X\right)\cdot P{C}_{m}\left(X\right)}{{\sum }_{X\in\Omega }{PC}_{m}\left(X\right)}$$

where

23 $${S}_{L}\left(X\right)={S}_{PC}\left(X\right)\cdot {S}_{G}\left(X\right)$$

24 $${S}_{PC}\left(X\right)=\frac{2{PC}_{1}\left(X\right)\cdot P{C}_{2}\left(X\right)+{T}_{1}}{P{C}_{1}^{2}\left(X\right)+P{C}_{2}^{2}\left(X\right)+{T}_{1}}$$

25 $${S}_{G}\left(X\right)=\frac{2{G}_{1}\left(X\right)\cdot {G}_{2}\left(X\right)+{T}_{2}}{{G}_{1}^{2}\left(X\right)+{G}_{2}^{2}\left(X\right)+{T}_{2}}$$

where ${T}_{1}$ and ${T}_{2}$ denote constants. Here, ${T}_{1}=0.85, {T}_{2}=160$. $\Omega$ is the whole space of image. $G$ is the gradient of image, defined as:

26 $$G=\sqrt{{G}_{x}^{2}+{G}_{y}^{2}}$$

$PC$ represents the phase consistency, defined as:

27 $$PC\left(X\right)=\frac{E\left(X\right)}{\varepsilon +\sum_{n}{A}_{n}\left(x\right)}$$

where ${A}_{n}\left(x\right)$ denotes $n$ order amplitude, $E\left(X\right)$ represents $n$ order response vector level at position $X$. $\varepsilon$ represents a small positive constant. Obviously, $FSIM$ closes to 1 for a well-segmented result and equals to 0 for the worst-segmented result.

### Experiments on synthetic images

**Figure 2:**
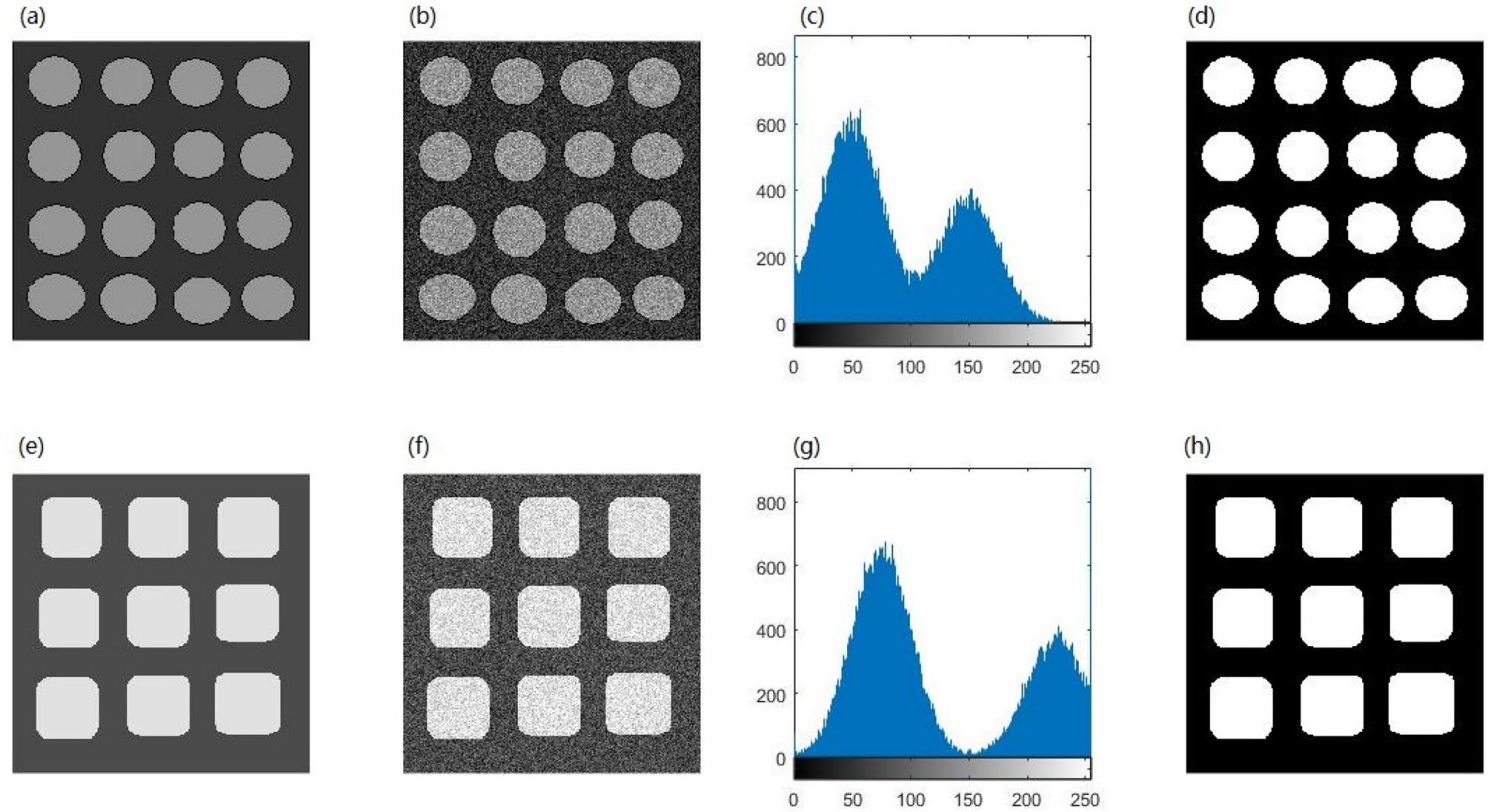
Two examples of synthetic images. (**a**) “Circles” image. (**b**) Noised “Circles” image. (**c**) Histogram of the noised “Circles” image. (**d**) Ground-truth image of noised “Circles”. (**e**) “Squares” image. (**f**) Noised “Squares” image. (**g**) Histogram of the noised “Squares” image. (**h**) Ground-truth image of noised “Squares”.

Synthetic images are perfect for testing the image thresholding algorithm because their optimal threshold values can be obtained manually^31^. Figure 2 shows two original synthetic images with $256\times 256$ pixels, which named as “Circles” and “Squares” [Fig. 2a,e], respectively. In Fig. 2a, we place some circles (their gray level is 150) on a darker background (gray level is 50). Figure 2b shows a noisy image of Fig. 2a with Gaussian noise, and Fig. 2c shows the histogram of Fig. 2b. Figure 2d shows the ground-truth image of Fig. 2b. In Fig. 2e, we place some squares (their gray level is 225) on a darker background (gray level is 75). Figure 2f shows a noisy image of Fig. 2e with Gaussian noise, and Fig. 2g shows the histogram of Fig. 2f. Figure 2h shows the ground-truth image of Fig. 2f.

For the synthetic “Circles” image, the optimal threshold value, which was calculated manually based on the ground-truth image, is 108. The threshold values, $MEs$, $NUs$ and $FSIMs$ obatined using the seven thresholding methods are listed in Table 1. The best values in terms of $MEs$, $NUs$ and $FSIMs$ are highlighted in bold. Among the seven thresholding methods, the threshold value obtained by WPWLPT is the closest to the optimal threshold value. It equals 110, and that the $ME$, $NU$ and $FSIM$ are equal to 0.0049, 0.0837 and 0.8103, respectively. The threshold value obtained using the OTSU method is 102. Its $ME$, $NU$ and $FSIM$ are equal to 0.0120, 0.1025 and 0.7998, respectively. The threshold value obtained using the KSW method is 82. Its $ME$, $NU$ and $FSIM$ are equal to 0.2650, 0.2208 and 0.6425, respectively. The threshold value obtained using the CHPSO_otsu method is 102. Its $ME$, $NU$ and $FSIM$ are equal to 0.0120, 0.1025 and 0.7998, respectively. The threshold value obtained using the CHPSO_ksw method is 84. Its $ME$, $NU$ and $FSIM$ are equal to 0.2595, 0.2172 and 0.6652, respectively. The threshold value obtained using the GLLV method is 101. Its $ME$, $NU$ and $FSIM$ are equal to 0.0156, 0.1368 and 0.7921, respectively. And the threshold value obtained using the GABOR method is 105. Its $ME$, $NU$ and $FSIM$ are equal to 0.0098, 0.1027 and 0.8024, respectively.

**Table 1:** The threshold values, $MEs$, $NUs$ and $FSIMs$ of the seven thresholding methods on the synthetic “Circles” image.

	OTSU	KSW	CHPSO_otsu	CHPSO_ksw	GLLV	GABOR	WPWLPT
Threshold value	102	82	102	84	101	105	110
$ME$	0.0120	0.2650	0.0120	0.2595	0.0156	0.0098	**0.0049**
$NU$	0.1025	0.2208	0.1025	0.2172	0.1368	0.1027	**0.0837**
$FSIM$	0.7998	0.6425	0.7998	0.6652	0.7921	0.8024	**0.8103**

The best results are highlighted in bold.

For the synthetic “Squares” image, the optimal threshold value, which was calculated manually based on the ground-truth image, is 153. The threshold values, $MEs$, $NUs$ and $FSIMs$ obatined using the seven thresholding methods are listed in Table 2. The best values in terms of $ME$, $NU$ and $FSIM$ are highlighted in bold. Among the seven thresholding methods, the threshold value obtained by WPWLPT was also the closest to the optimal threshold value. It equals 152, and that the $ME$, $NU$ and $FSIM$ are equal to 0.0018, 0.0386 and 0.8198, respectively. The threshold value obtained using the OTSU method is 147. Its $ME$, $NU$ and $FSIM$ are equal to 0.0020, 0.0405 and 0.8002, respectively. The threshold value obtained using the KSW method is 108. Its $ME$, $NU$ and $FSIM$ are equal to 0.0606, 0.1982 and 0.6512, respectively. The threshold value obtained using the CHPSO_otsu method is 148. Its $ME$, $NU$ and $FSIM$ are equal to 0.0019, 0.0401 and 0.8076, respectively. The threshold value obtained using the CHPSO_ksw method is 110. Its $ME$, $NU$ and $FSIM$ are equal to 0.0618, 0.1884 and 0.6725, respectively. The threshold value obtained using the GLLV method is 150. Its $ME$, $NU$ and $FSIM$ are equal to 0.0018, 0.0398 and 0.8178, respectively. And the threshold value obtained using the GABOR method is 146. Its $ME$, $NU$ and $FSIM$ are equal to 0.0021, 0.0403 and 0.8104, respectively.

**Table 2:** The threshold values, $MEs$, $NUs$ and $FSIMs$ of the seven thresholding methods on the synthetic “Squares” image.

	OTSU	KSW	CHPSO_otsu	CHPSO_ksw	GLLV	GABOR	WPWLPT
Threshold value	147	108	148	110	150	146	152
$ME$	0.0020	0.0606	0.0019	0.0618	0.0018	0.0021	**0.0018**
$NU$	0.0405	0.1982	0.0401	0.1884	0.0398	0.0403	**0.0386**
$FSIM$	0.8002	0.6605	0.8076	0.6725	0.8178	0.8104	**0.8198**

The best results are highlighted in bold.

As can be seen from the results of the threshold values, $MEs$, $NUs$ and $FSIMs$, it is clear that:

For the synthetic “Circles” image, the threshold values of KSW and CHPSO_ksw are 82 and 84, respectively. They are almost worthless threshold values, because of them far from the optimal threshold (108). In contrast, the threshold values of OTSU, CHPSO_otsu, GLLV, GABOR and WPWLPT are 102, 102, 101, 105, 110, respectively, which are reasonable threshold values due to their near to the optimal value. Especially, the threshold value of our WPWLPT is only 2 larger than the optimal threshold. The results in terms of $MEs$, $NUs$ and $FSIMs$ also reveal that our WPWLPT yields the best results. The $MEs$ and $NUs$ provided by the KSW and CHPSO_ksw methods were so higher, and the $FSIMs$ were so lower than other methods. While OTSU, CHPSO_otsu, GLLV, GABOR and WPWLPT can obtain reasonable results, especially our WPWLPT method which obtains the minimum $ME$, $NU$ and the maximum $FSIM$ values.

For the synthetic “Squares” image, the threshold values of KSW and CHPSO_ksw are 108 and 110, respectively. These are almost worthless threshold values too, because they are from the optimal threshold (153). In contrast, the threshold values of OTSU, CHPSO_otsu, GLLV, GABOR and WPWLPT are 147, 148, 150, 146, 152, respectively, which are reasonable threshold values because they are close to the optimal value. Especially, the threshold value of our WPWLPT is only 1 less than the optimal threshold. The results in terms of $MEs$, $NUs$ and $FSIMs$ also reveal that our WPWLPT yields the minimum $ME$, $NU$ and the maximum $FSIM$ values, which were best results among all the seven thresholding methods.

**Figure 3:**
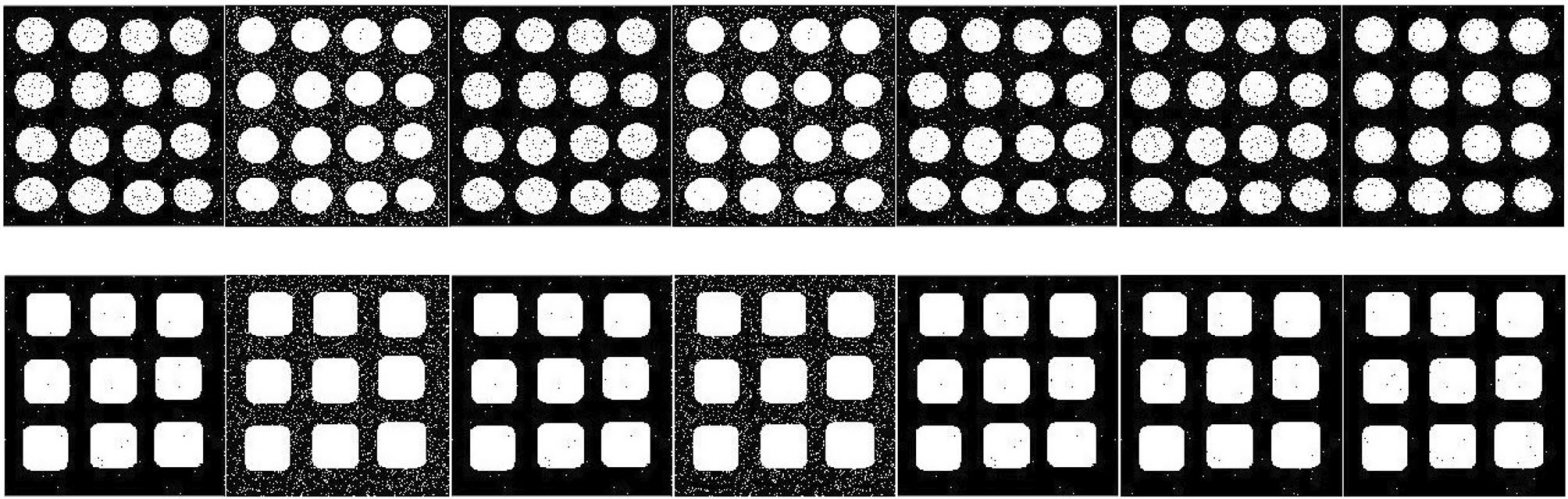
Thresholding segmentation results of the noised synthetic image by using different methods. For each experimental synthetic image, the results of OTSU, KSW, CHPSO_otsu, CHPSO_ksw, GLLV, GABOR and WPWLPT methods are displayed side by side from left to right.

Figure 3 provides a visual comparison between the thresholding results obtained by the OTSU, KSW, CHPSO_otsu, CHPSO_ksw, GLLV, GABOR and the proposed WPWLPT methods. As can be seen from Fig. 3, the KSW and CHPSO_ksw methods obtained almost unvalued results because their segmented images had obvious noise (see Fig. 3, the second and fourth images of each row). In contrast, all the OTSU, CHPSO_otsu, GLLV, GABOR and WPWLPT methods segmented a cleaner image because the threshold values they obtained were close to the optimal threshold value. Furthermore, by zooming in Fig. 3, we can easily observe that the WPWLPT method gives the clearest segmentation results compared with the OTSU, CHPSO_otsu, GLLV and GABOR methods, because it has the least residual noise.

### Experiments on NDT images

**Figure 4:**
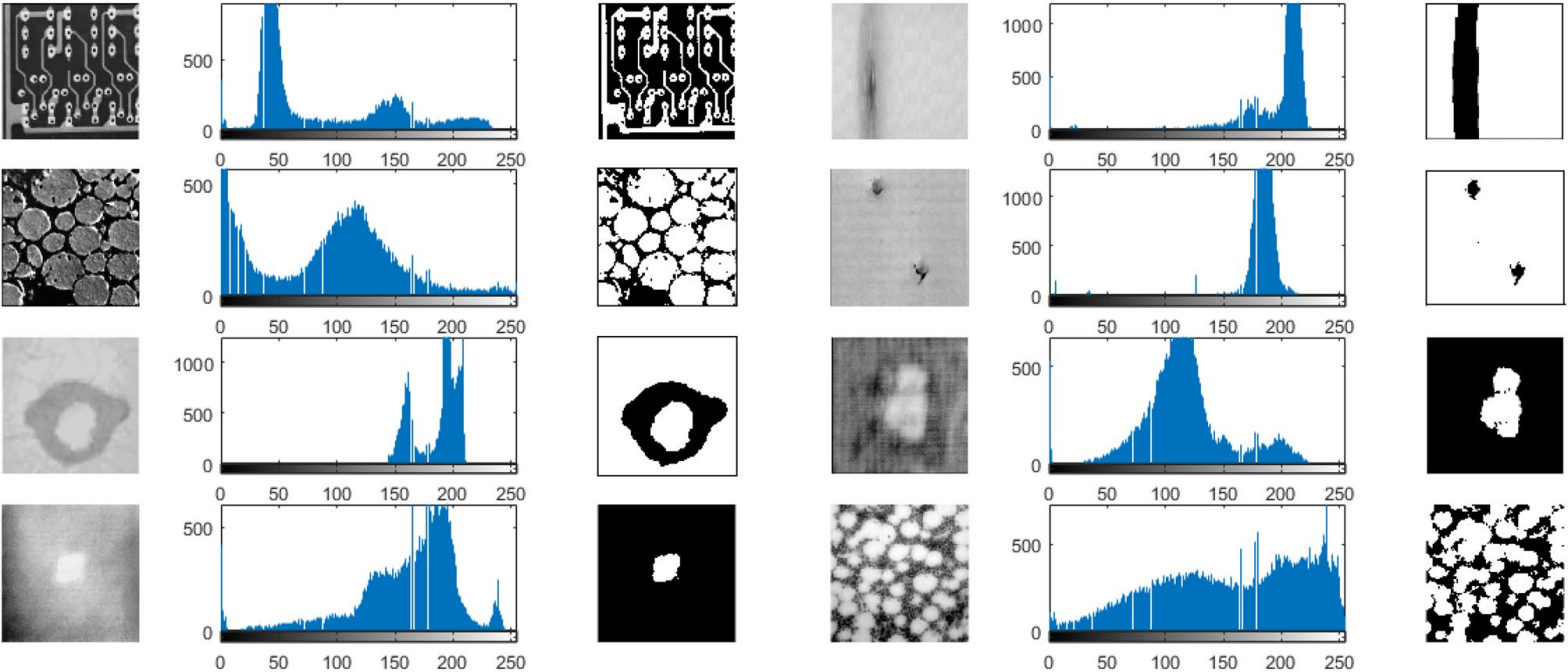
The experimental NDT images, their histograms and ground-truth images.

**Figure 5:**
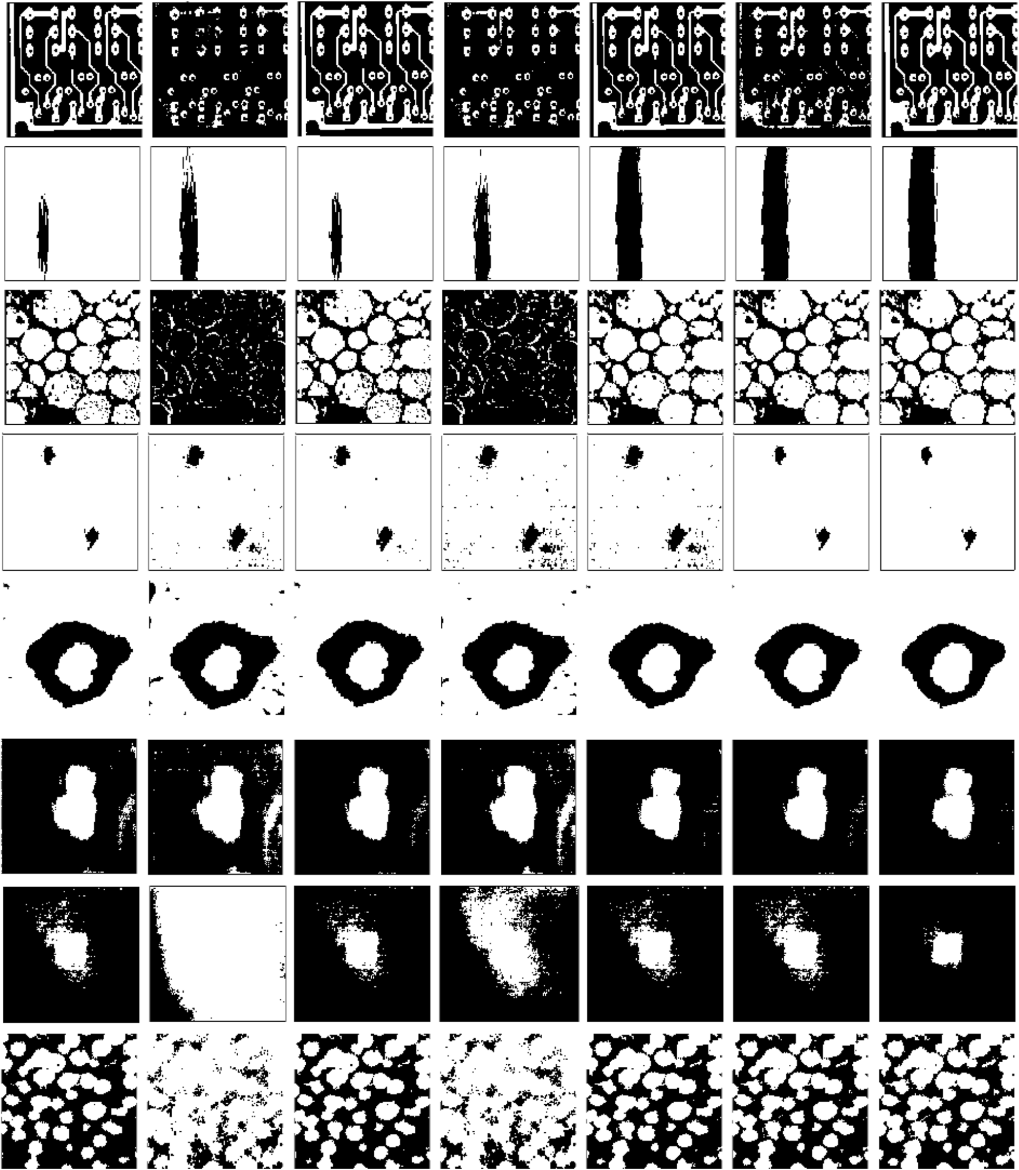
Thresholding segmentation results by using different methods. For each experimental image, the results of OTSU, KSW, CHPSO_otsu, CHPSO_ksw, GLLV, GABOR and WPWLPT methods are displayed side by side from left to right.

The NDT images are also ideal for testing the image thresholding algorithm because their ground-truth images can be obtained directly. In this part, eight NDT images were used to assess the performance of the WPWLPT. They are “PCB”, “defective tile”, “material structure”, “fuselage material”, “eddy current”, “ultrasonic”, “GFRP”and “bonemarr”. All the above eight images, their histograms and ground-truth images are shown in Fig. 4. The thresholding segmentation results obtained using the reference thresholding methods and WPWLPT are shown in Fig. 5.

Tables 3, 4 and 5 show the $MEs$, $NUs$ and $FSIMs$ of the different thresholding methods, respectively. ${\varpi }_{ME}$,${\varpi }_{NU}$ and ${\varpi }_{FSIM}$ represent the average of $MEs$, $NUs$ and $FSIMs$ respectively. The best results are highlighted in bold.

**Table 3:** $MEs$ of the different thresholding methods.

Image	OTSU	KSW	CHPSO_otsu	CHPSO_ksw	GLLV	GABOR	WPWLPT
PCB	0.0320	0.2170	0.0376	0.1828	0.0380	0.0886	**0.0176**
Defective tile	0.1478	0.1668	0.1337	0.1576	0.0297	0.0249	**0.0198**
Material structure	0.0895	0.6176	0.0626	0.5562	**0.0503**	0.0536	**0.0528**
Fuselage material	0.0207	0.0711	0.0257	0.0942	0.0728	**0.0199**	**0.0199**
Eddy current	0.0253	0.0407	0.0229	0.0402	0.0209	0.0206	**0.0193**
Ultrasonic	0.0405	0.1021	0.0356	0.0829	0.0215	0.0275	**0.0203**
GFRP	0.0855	0.4898	0.0765	0.3986	0.0945	0.1008	**0.0669**
Bonemarr	0.1021	0.3256	0. 1038	0. 3338	0.0976	0.0958	**0.0921**
${\varpi }_{ME}$	0.0679	0.2538	0.0623	0.2308	0.0532	0.0540	**0.0386**

The best results are highlighted in bold.

**Table 4:** $NUs$ of the different thresholding methods.

Image	OTSU	KSW	CHPSO_otsu	CHPSO_ksw	GLLV	GABOR	WPWLPT
PCB	0.1228	0.6725	0.1228	0.5265	**0.1208**	0.1492	0.1225
Defective tile	0.2037	0.1583	0.2012	0.1526	0.0988	0.0968	**0.0935**
Material structure	0.1846	0.7036	0.1676	0.6092	0.1105	**0.1025**	0.1078
Fuselage material	0.0898	0.1678	0.1102	0.1876	0.1798	0.0812	**0.0724**
Eddy current	0.0675	0.1239	0.0598	0.0960	0.0588	0.0585	**0.0580**
Ultrasonic	0.1036	0.1478	0.0983	0.1421	0.0827	0.0841	**0.0784**
GFRP	0.0858	0.7879	0.0838	0.6035	0.0980	0.1092	**0.0837**
Bonemarr	0.1051	0.3465	0.1011	0.3379	0.0982	0.0976	**0.0921**
${\varpi }_{NU}$	0.1204	0.3885	0.1181	0.3319	0.1060	0.0974	**0.0886**

The best results are highlighted in bold.

**Table 5:** $FSIMs$ of the different thresholding methods.

Image	OTSU	KSW	CHPSO_otsu	CHPSO_ksw	GLLV	GABOR	WPWLPT
PCB	0.6882	0.5864	0.7005	0.6643	0.6801	0.5948	**0.7013**
Defective tile	0.5845	0.6005	0.5801	0.5992	0.6716	0.6895	**0.7052**
Material structure	**0.7112**	0.5028	0.6708	0.5802	0.6786	0.6882	0.6931
Fuselage material	0.7128	0.7466	0.7209	0.7375	0.7314	0.7684	**0.7690**
Eddy current	0.7088	0.7002	0.7108	0.7103	0.7110	**0.7116**	0.7112
Ultrasonic	0.6402	0.6327	0.6508	0.6388	0.6851	0.6816	**0.7013**
GFRP	0.7270	0.6036	0.7178	0.6616	0.7104	0.7037	**0.7294**
Bonemarr	0.7008	0.6018	0.7045	0.6055	0.7125	0.7101	**0.7237**
${\varpi }_{FISM}$	0.6842	0.6218	0.6820	0.6497	0.6976	0.6935	**0.7168**

The best results are highlighted in bold.

Because of length limits, we only analyzed four NDT images. They are the “PCB”, “material structure”, “eddy current” and “GFRP”.

For “PCB” image, the $ME$ and $FSIM$ values obtained by WPWLPT method are optimal, while the $NU$ value is inferior to GLLV method only. This is reasonable because they focused on different aspects of measurement. $ME$ represents the percentage of background pixels incorrectly classified to the foreground, or vice versa, $FSIM$ focuses on the texture, shape and other features, while $NU$ judges the intrinsic quality of the segmented areas. The worst results are obtained using the KSW method. Its $ME$, $NU$ and $FSIM$ values are equal to 0.2170, 0.6725 and 0.5864, respectively. Obviously, compared with other methods, its $ME$ and $NU$ values are too high and $FSIM$ value is too low, making the results worthless. A visual comparison, as shown in Fig. 5, shows that the OTSU, CHPSO_otsu, GLLV and WPWLPT methods can segment better segmentation image. By comparison, the KSW, CHPSO_ksw and GABOR methods segmented valueless results because they misclassify lots of foregrounds as backgrounds (see Fig. 5, first row, second, fourth and sixth images, they can’t distinguish the backgrounds and printed circuit board, especially the second image).

For “material structure” image, GLLV, GABOR and OTSU yield the best $ME$ $NU$ and $FSIM$ values, respectively. However, WPWLPT yields the closest values of $ME$, $NU$ and $FSIM$ values to the best. The worst results are obtained using the KSW method. Its $ME$, $NU$ and $FSIM$ equal to 0.6176, 0.7036 and 0.5028, respectively. Obviously, its $ME$ and $NU$ values are too high and $FSIM$ value is too low, so the results obtained are worthless. The visual comparison, as can be seen from Fig. 5, we can also discover that the OTSU, CHPSO_otsu, GLLV and WPWLPT methods can segment better segmentation image. By comparison, the KSW and CHPSO_ksw methods segment almost an unvalued segmentation image because they misclassify lots of foregrounds as backgrounds (see Fig. 5, third row, second and fourth image, the whole image looks black).

For “eddy current” image, the $ME$ and $NU$ values obtained by WPWLPT method are optimal, while the $FISM$ value is inferior to GABOR method only. The worst results are obtained using the KSW method. Its $ME$, $NU$ and $FSIM$ values are equal to 0.0407, 0.1239 and 0.7002, respectively. Similar to the results of the KSW method, the CHPSO_ksw method also yielded poor results for $ME$, $NU$ and $FSIM$ values. The visual comparison, as can be seen from Fig. 5, we can also discover that the GLLV, GABOR and WPWLPT methods can segment better segmentation images. By comparison, the KSW and CHPSO_ksw methods segment a low-value segmentation image because they misclassify some backgrounds as foregrounds (see Fig. 5, fifth row, second and fourth images, some black shadows appeared in the segmentation image).

For “GFRP” image, all the $ME$, $NU$ and $FISM$ values obtained by WPWLPT method are optimal. The worst results are also obtained by the KSW method. Its $ME$, $NU$ and $FSIM$ values are equal to 0.4898, 0.7879 and 0.6036, respectively. Obviously, the $ME$ (0.7879!) is close to 1 which corresponding to the worst case. The results obtained by CHPSO_ksw method are also valueless due to their higher $ME$, $NU$ values and lower $FSIM$ values. A visual comparison, as can be seen from Fig. 5, shows that the OTSU, CHPAO_otsu, GLLV, GABOR and WPWLPT methods can segment better segmentation images. By comparison, the KSW and CHPSO_ksw methods segmented valueless results because they misclassify lots of backgrounds as foregrounds (see Fig. 5, seventh row, second and fourth images, it's impossible to distinguish between foregrounds and backgrounds).

### Experiments on a set of benchmark images

**Figure 6:**
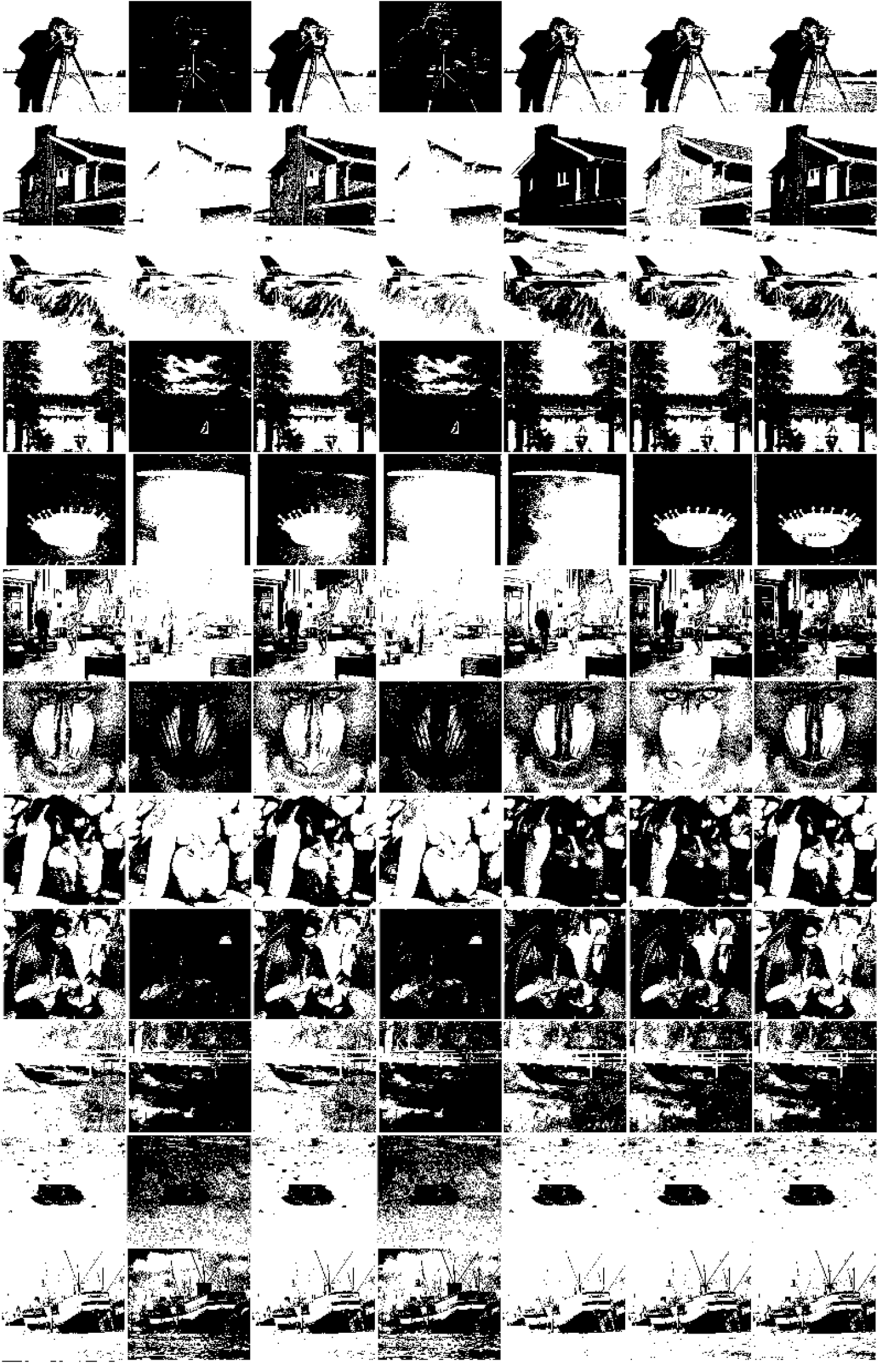
Thresholding segmentation results by using different methods. For each experimental image, the results of OTSU, KSW, CHPSO_otsu, CHPSO_ksw, GLLV, GABOR and WPWLPT methods are displayed side by side from left to right.

A set of benchmark images belonging to the Image Processing Standard Database and USC-SIPI Image Database, which contain 12 Gy images. For brevity, we give 12 images here, “cameraman”, “house”, “jetplane”, “lake”, “milkdrop”, “livingroom”, “mandril”, “peppers”, “pirate”, “walkbridge”, “tank”, and “boat”, all in uncompressed tif or tiff format and of the same $512\times 512$ size. The thresholding segmentation results of the corresponding twelve images obtained by the reference thresholding methods and WPWLPT are shown row by row from top to bottom in Fig. 6. Tables 6 and 7 show the $NUs$, and $FSIMs$ of different thresholding methods, respectively. ${\varpi }_{NU}$, and ${\varpi }_{FSIM}$ represent the average of $MEs$, and $FSIMs$ respectively. The best results are highlighted in bold. It should be noted that, we did not employ $ME$ to measure the quality of thresholding methods experiment on these images, because the ideal thresholded or ground-truth images cannot be acquired.

**Table 6:** $NUs$ of the different thresholding methods.

Image	OTSU	KSW	CHPSO_otsu	CHPSO_ksw	GLLV	GABOR	WPWLPT
Cameraman	0.1245	0.8587	0.1236	0.8326	0.6207	0.1828	**0.1096**
House	0.1702	0.6299	0.1825	0.6285	0.2052	0.3116	**0.1624**
Jetplane	0.1852	0.2225	0.1825	0.2208	0.1651	**0.1603**	0.1644
Lake	0.0718	0.3499	**0.0707**	0.3423	0.0737	0.0726	0.0720
Milkdrop	0.1027	0.7182	0.1083	0.7174	0.7083	0.0486	**0.0428**
Livingroom	0.0988	0.2059	0.0976	0.1895	**0.0889**	0.1025	0.1122
Mandril	0.0798	0.1821	0.0788	0.1879	**0.0668**	0.1356	0.0672
Peppers	0.0836	0.1328	0.0828	0.1330	0.1008	0.1012	**0.0728**
Pirate	**0.1028**	0.6738	**0.1028**	0.6689	0.1106	0.1274	0.1031
Walkbridge	0.1324	0.1228	0.1302	0.1225	0.1138	0.1108	**0.1102**
Tank	0.1106	0.2807	0.1098	0.2321	0.1048	0.1017	**0.1009**
Boat	0.0806	0.1489	0.0826	0.1478	0.0854	0.0788	**0.0725**
${\varpi }_{NU}$	0.1119	0.3772	0.1127	0.3686	0.2037	0.1278	**0.0922**

The best results are highlighted in bold.

**Table 7:** $FSIMs$ of the different thresholding methods.

Image	OTSU	KSW	CHPSO_otsu	CHPSO_ksw	GLLV	GABOR	WPWLPT
Cameraman	0.6978	0.4684	0.6988	0.6223	0.6529	0.6925	**0.7168**
House	**0.7427**	0.2323	0.7207	0.2416	0.7022	0.6628	0.7418
Jetplane	0.7375	0.7026	0.7399	0.7101	0.7842	**0.7883**	0.7865
Lake	0.7569	0.6004	0.7569	0.6011	**0.7588**	0.7535	0.7518
Milkdrop	0.7301	0.3872	0.7315	0.3885	0.4018	0.8178	**0.8288**
Livingroom	**0.7498**	0.6585	0.7467	0.6568	0.7005	0.7269	0.7326
Mandril	0.8280	0.7326	**0.8396**	0.7288	0.8288	0.7998	0.8328
Peppers	**0.8568**	0.7358	**0.8568**	0.7384	0.8113	0.8012	**0.8568**
Pirate	0.7359	0.3684	0.7402	0.3744	0.7227	0.7129	**0.7625**
Walkbridge	0.6339	0.7002	0.6376	0.7083	0.7367	**0.7452**	**0.7452**
Tank	0.8515	0.7598	0.8520	0.7678	0.8598	0.8692	**0.8712**
Boat	0.7998	0.7288	0.7962	0.7312	0.8010	0.8056	**0.8134**
${\varpi }_{FISM}$	0.7601	0.5896	0.7597	0.6058	0.7301	0.7646	**0.7867**

The best results are highlighted in bold.

As shown in Fig. 6, Tables 6 and 7, it can be observed that:

For most of the tested images, the values of $NU$ and $FISM$ obtained by WPWLPT are the lowest. Specifically, for the “cameraman”, “house”, “milkdrop”, “peppers”, “walkbridge”, “tank” and “boat” images, the proposed WPWLPT method can obtain the lowest $NU$ values. For the “pirate” image, OTSU obtains the lowest $NU$ values. For the “lake” and “pirate” images, CHPSO_otsu obtains the lowest $NU$ values. For the “livingroom” and “mandril” images, GLLV obtains the lowest $NU$ values. For the “jetplane” image, GABOR obtains the lowest $NU$ values. In terms of $FISM$, the proposed method are similar. It obtains the highest $FISM$ values in the “cameraman”, “milkdrop”, “peppers”, “pirate”, “walkbridge”, “tank” and “boat” images. Although the WPWLPT method did not obtain the lowest $NU$ values and highest $FISM$ values for all 12 test images, the average value of $NUs$ and $FISMs$ were the best among all seven thresholding methods. Specifically, the ${\varpi }_{NU}$ values of OTSU, KSW, CHPSO_otsu, CHPSO_ksw, GLLV, GABOR and WPWLPT are equal to 0.1119, 0.3772, 0.1127, 0.3686, 0.2037, 0.1278 and 0.0992, respectively. The ${\varpi }_{FISM}$ values of OTSU, KSW, CHPSO_otsu, CHPSO_ksw, GLLV, GABOR and WPWLPT equal to 0.7601, 0.5896, 0.7597, 0.6058, 0.73014, 0.7646 and 0.7867, respectively. It means that the ${\varpi }_{NU}$ value obtain by our method outperforms the OTSU, KSW, CHPSO_otsu, CHPSO_ksw, GLLV and GABOR methods by 1.27%, 27.80%, 1.35%, 26.94%, 10.45% and 2.87% respectively, while the ${\varpi }_{FISM}$ value outperforms the OTSU, KSW, CHPSO_otsu, CHPSO_ksw, GLLV and GABOR methods by 2.66%, 19.71%, 2.69%, 18.09%, 5.66% and 2.20% respectively.

From the above analysis, we can assert that the WPWLPT method can calculate better ${\varpi }_{NU}$ and ${\varpi }_{FISM}$ values in comparision with other reference methods. This result demonstrates the stability and accuracy of the proposed method.

### $mIou$ values

$mIoU$ is the more widely used objective metric for the task of image segmentation. It is defined as:

28 $$mIoU=\frac{1}{k+1}\sum_{i=0}^{k}\frac{TP}{FN+FP+TP}$$

where $k$ is the number of classes, $TP, FN and FP$ denote true positives, false positives and false positives, respectively. We employ $mIoU$ to objectively evaluate synthetic and NDT images because they have ground-truth images. The $mIoUs$ obtained by different thresholding methods are listed in Table 8. ${\varpi }_{mIoU}$ represents the average of $mIoUs$. The best results are highlighted in bold.

**Table 8:** $mIoUs$ (%) of the different thresholding methods.

Image	OTSU	KSW	CHPSO_otsu	CHPSO_ksw	GLLV	GABOR	WPWLPT
Circles	86.9	66.2	86.0	66.6	88.6	89.1	**89.6**
Squares	87.8	79.8	85.8	84.4	**89.8**	**89.8**	**89.8**
PCB	82.3	70.5	84.7	73.5	84.7	82.0	**86.5**
Defective tile	59.7	75.0	60.6	71.6	85.4	85.8	**86.3**
Material structure	72.8	38.2	75.0	44.4	80.7	80.4	**83.4**
Fuselage material	82.3	76.2	82.8	74.3	76.0	83.3	**84.3**
Eddy current	85.8	84.4	86.0	84.5	87.6	88.1	**88.3**
Ultrasonic	78.7	71.8	82.0	73.4	87.1	86.6	**87.2**
GFRP	75.9	35.7	76.7	48.1	75.2	74.6	**79.3**
Bonemarr	79.9	54.0	79.8	53.3	81.2	80.5	**81.2**
${\varpi }_{mIoU}$	78.7	65.8	79.5	68.2	83.3	83.7	**85.3**

The best results are highlighted in bold.

As shown in Table 8, in most test images, the values of $mIoU$ obtained by WPWLPT are the highest. Specifically, for the “Circles”, “PCB”, “defective tile “, “material structure”, “fuselage material “, “eddy current”, “ultrasonic” and “GFRP” images, the proposed WPWLPT method can obtain the highest $mIoU$ values. For the “, “Squares” image, GLLV, GABOR and WPWLPT both obtained the highest $mIoU$ value. For the “bonemarr” image, GLLV and WPWLPT both got the highest $mIoU$ values.

**Figure 7:**
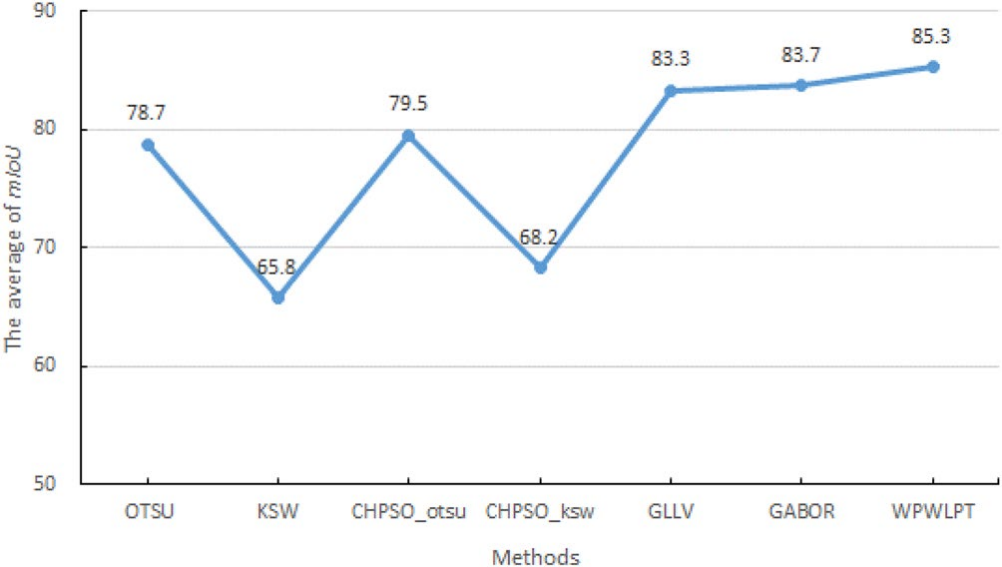
The average $mIoUs$ (%) of different methods.

Figure 7 depicts the average values of $mIoU$ for the synthetic and NDT images. As shown in Fig. 7, our method has been improved to varying degrees on average $mIoU$ compared with other methods. Specifically, the ${\varpi }_{mIoU}$ values of OTSU, KSW, CHPSO_otsu, CHPSO_ksw, GLLV, GABOR and WPWLPT are equal to 78.7%, 65.8%, 79.5%, 68.2%, 83.3%, 83.7% and 85.3%, respectively. It means that the ${\varpi }_{mIoU}$ value obtain by our method outperforms the OTSU, KSW, CHPSO_otsu, CHPSO_ksw, GLLV and GABOR methods by 6.0%, 19.5%, 5.8%, 17.1%, 2.0% and 1.6%, respectively.

### Discussion

Based on the above analysis of the experimental results of synthetic, NDT and a benchmark images, we find that:

1. The proposed WPWLPT method obtained the best segmentation performance for most images. In addition, our method can yields the lowest ${\varpi }_{ME}$ and ${\varpi }_{NU}$, the highest ${\varpi }_{FISM}$ and ${\varpi }_{mIoU}$ on all the synthetic, NDT and the benchmark of images. Specifically, for the two synthetic images, the ${\varpi }_{ME}$, ${\varpi }_{NU}$, and ${\varpi }_{FISM}$ of WPWLPT equal to 0.0034, 0.0612 and 0.8151, respectively. The ${\varpi }_{ME}$ of WPWLPT outperforms the competing methods by 0.0037 to 0.1595, the ${\varpi }_{NU}$ outperforms the competing methods by 0.0102 to 0.1484, and the ${\varpi }_{FISM}$ outperforms the competing methods by 0.0101 to 0.1636. For the eight NDT image, the ${\varpi }_{ME}$, ${\varpi }_{NU}$, and ${\varpi }_{FISM}$ of WPWLPT equal to 0.0386, 0.0886 and 0.7168, respectively. The ${\varpi }_{ME}$ of WPWLPT outperforms the competing methods by 0.0146 to 0.2152, the ${\varpi }_{NU}$ outperforms the competing methods by 0.0088 to 0.2999, and the ${\varpi }_{FISM}$ outperforms the competing methods by 0.0192 to 0.0950. For the benchmark of twelve images, the ${\varpi }_{NU}$, and ${\varpi }_{FISM}$ of WPWLPT equal to 0.0992, and 0.7867, respectively. The ${\varpi }_{NU}$ outperforms the competing methods by 0.0127 to 0.2780, and the ${\varpi }_{FISM}$ outperforms the competing methods by 0.0220 to 0.1971. For all the ten synthetic and NDT images, The ${\varpi }_{mIoU}$ of WPWLPT equals to 85.3%, which outperforms the competing methods by 1.6% to 19.5%. These experimental results well demonstrate the effectiveness and robustness of the proposed WPWLPT method.
2. From a visual perspective (Figs. 3, 5 and 6), although for some images, our method does not achieve the best segmentation effectiveness, it can obtain acceptable or close to the best results, which also shows the stability of our method.
3. OTSU is a traditional method, that exhibits high stability and accuracy. It outperformed most of the other methods except for ours.
4. Although our method works well for most images, it doesn’t yield best performance on “material structure”, “lake”, “milkdrop” et al. (For these three images, all the ${\varpi }_{ME}$, ${\varpi }_{NU}$, ${\varpi }_{FISM}$ or ${\varpi }_{mIoU}$ values are not optimal). The possible reason is the boundary information, which plays a crucial role in our proposed method is not obvious.
5. The KSW and CHPSO_ksw methods are the two worst performing methods. The Kapur based method is 1D entropy without considering other information. Obviously, GLLV and GABOR methods are superior to Kapur based method because they introduce other information such as gradient magnitude, texture and contour, etc. This also gave us inspiration to introduce other information into our method, which is our next step. This also gives us a clue to introduce other information into our method, and contour information is a potential choice. Moreover, it can be seen as our future work.

### Running time

In addition to the qualitative and quantitative assessment, the running time of the thresholding method is another important evaluation. Table 9 reports the average running time obtained by different threshold methods for the above 22 test images. All the experiments are running on the DELL notebook with Intel(R) Core (TM) i5-4300U CPU @ 1.90 GHz 2.50GHZ, 16 GB memory. The running environment is Matlab (R2015b). As shows in Table 9, the proposed WPWLPT method takes approximately the same amount of time as the GLLV method. It is superior to the GABOR method but inferior to 1D methods, such as OTSU, KSW, CHPSO_otsu and CHPSO_ksw. Although our method is slower than OTSU, KSW etc., the running time is completely acceptable in many applications. In addition, our WPWLPT method is still highly competitive because of its superior effectiveness and robustness.

**Table 9:** The average running times (s) of different thresholding methods for all the 22 test images.

Method	OTSU	KSW	CHPSO_otsu	CHPSO_ksw	GLLV	GABOR	WPWLPT
Average running time (s)	0.4580	0.5000	0.4280	0. 4890	6.8500	65.7500	6.9500

## Conclusions

In this study, a new image bi-level thresholding method is proposed. The method first obtains the boundaries for the foreground and background in the image using a weighted Parzen-window to describe the gray level distribution status. Secondly, the image thresholding problem can be transformed into the problem of solving a linear programming problem for computing the coefficient values of the weighted Parzen-window. By solving the problem of linear programming, we determine the threshold. In the experiment, we used two synthetic, eight NDT and a benchmark of twelve testing images, which have different histogram types, to evaluate the quality of the proposed image thresholding method. The measurement of visual and quantitative results demonstrates that our proposed method, compared with the OTSU, KSW, CHPSO_otsu, CHPSO_ksw, GLLV and GABOR methods, can achieve better effectiveness and robustness. In the future, as an extension of this work, we will embed other information, such as texture, contour etc. in WPWLPT to enhance its performance, and extend the method to the problem of multilevel thresholding.

## Data Availability

The data underlying this article will be shared on reasonable request to the corresponding author.
